# Relationships between attachment insecurity, beliefs about the self and others, paranoia, and social functioning across the psychosis continuum

**DOI:** 10.1111/bjc.70027

**Published:** 2026-01-21

**Authors:** Pilar de‐la‐Higuera‐Gonzalez, Carsten Allefeld, Alejandro de‐la‐Torre‐Luque, Ana Isabel Guillén, Marina Díaz‐Marsá, Anne‐Kathrin Fett

**Affiliations:** ^1^ Department of Personality, Assessment and Clinical Psychology, Faculty of Psychology Universidad Complutense de Madrid (UCM) Madrid Spain; ^2^ Foundation for the Biomedic Investigation HCSC Health Investigation Institute Hospital Clinico San Carlos (IdISSC) Madrid Spain; ^3^ Department of Psychology and Neuroscience School of Health & Medical Sciences, City St George's, University of London London UK; ^4^ Department of Legal Medicine, Psychiatry and Pathology, Faculty of Medicine Universidad Complutense de Madrid (UCM) Madrid Spain; ^5^ Biomedical Research Networking Consortium for Mental Health (CIBERSAM ISCII) Madrid Spain; ^6^ Department of Psychosis Studies, Institute of Psychiatry, Psychology and Neuroscience King's College London London UK

**Keywords:** Attachment, beliefs about others, experience sampling method, paranoia, psychosis, self‐beliefs, social engagement

## Abstract

**Objectives:**

Self‐beliefs and other‐regarding beliefs are related to attachment experiences and may contribute to paranoid beliefs and social functioning difficulties in psychosis. However, their relationships have not been examined jointly from an ecological perspective, while considering different degrees of psychosis risk.

**Design:**

Relationships between avoidant and anxious attachment, self‐beliefs and other‐regarding beliefs, paranoia, and social functioning in daily‐life were examined across the psychosis continuum of familial risk.

**Methods:**

The sample comprised 29 patients with non‐affective psychotic disorders (*M*_age_ = 39.07, *SD* = 9.91, 20.68% female), 17 first‐degree relatives (*M*_age_ = 37.36, *SD* = 13.86, 64.71% female) and 26 controls (*M*_age_ = 36.15, *SD* = 8.1, 34.6% female). Avoidant and anxious attachment were assessed with the Psychosis Attachment Measure. Self‐beliefs, beliefs about others, paranoia, and time spent alone were assessed for one week in participants’ daily lives using the Experience Sampling Method. Multilevel models were used to investigate cross‐sectional and temporal relationships between the variables.

**Results:**

Less positive self‐beliefs and beliefs about others were related to paranoia, but only self‐beliefs mediated the association between attachment insecurity and paranoia. People who were alone more frequently held less positive self‐beliefs and beliefs about others, and being alone at a specific point in time was related to less positive self‐beliefs. Attachment insecurity was unrelated to the amount of time spent alone.

**Conclusions:**

Less positive self‐beliefs and beliefs about others were related to higher paranoia levels and fewer social interactions in daily‐life. These associations were present in all groups, supporting their utility as cognitive treatment targets in diverse therapeutic contexts.

**Practitioner points**

- Self‐beliefs are related to attachment experiences and important therapeutic goals in the treatment of suspiciousness, paranoid delusions, and social isolation.
- Additionally, focusing on beliefs about others and on how they relate to suspiciousness and lack of social contact could support recovery by improving paranoid beliefs and social isolation.
- The therapeutic goals are applicable to people with clinical psychosis, but may also be suitable for non‐clinical populations who experience more subtle suspiciousness and paranoia.

## INTRODUCTION

Schizophrenia or other primary non‐affective psychotic disorders are characterised by positive and negative symptoms (World Health Organization, 2021) and sustained difficulties in social functioning (Caple et al., 2023; Pinkham et al., 2012; Velthorst et al., 2017). Social functioning is often operationalised as the frequency of social contact. Studies show that patients spend more time alone than their healthy relatives or controls (Fett et al., 2022) and patients with other psychiatric disorders (Giacco et al., 2016; Velthorst et al., 2017). Specific symptoms, such as paranoid delusions, may aggravate social difficulties (Bentall et al., 2009; Degnan et al., 2018). Paranoid delusions are persistent beliefs about malevolent intentions of others that entail high levels of self‐reference of others’ actions (Bentall et al., 2009; World Health Organization, 2021). Higher levels of paranoia in patients with psychotic disorders have been related to social impairment, feelings of loneliness, and social exclusion (Bell et al., 2023; Kesting et al., 2013; Lamster et al., 2017; Pinkham et al., 2016). Paranoid beliefs are also common in the general population, albeit to a lesser extent. Thus, paranoia can be considered as a dimensional continuum (Freeman et al., 2005; Hajdúk et al., 2019; Johns & van Os, 2001). In the general population, paranoid thoughts have been related to high levels of distress (Freeman et al., 2005; Garety et al., 2001), worse interpersonal functioning, and greater interpersonal sensitivity (Hajdúk et al., 2019; Meisel et al., 2018).

Cognitive models of psychosis suggest that paranoid delusions are a product of previous cognitive content and life experiences. When individuals face a potential external threat, a search of meaning is activated, and concurrently with high arousal and cognitive biases, previous self‐beliefs and beliefs about others influence the explanation to conform to the delusional explanation (Freeman et al., 2002). Existing evidence links lower levels of self‐esteem to higher paranoid beliefs in clinical and non‐clinical samples (Jones et al., 2010; Kesting et al., 2013; Kesting & Lincoln, 2013; Thewissen et al., 2008, 2011; Tiernan et al., 2014). Also, negative beliefs about others have been related to increased paranoia levels (Lamster et al., 2017; Sood et al., 2021). It has been proposed that relationships between self‐beliefs, beliefs about others, and paranoia may be understood as defences of the self, where paranoid beliefs emerge when a threat to the self is perceived and discrepancies between the actual and ideal self are increased. Paranoia may thus act to minimise this discrepancy through attributions of negative self‐views to others (Bentall et al., 1994); however, while this is a possible mechanism, it is a contested point (Murphy et al., 2018) and alternative explanations have been proposed (Bentall et al., 2001).

Self‐beliefs and beliefs about others are formed throughout social development, integration, and revision of different representations about bonds with others, i.e., our attachments (Hazan & Shaver, 1987). When caregivers are available and sensitive, secure attachment bonds are developed, but when they are not, no sense of security can be formed and insecure attachments result (Ainsworth et al., 1978; Bowlby, 1969; Hazan & Shaver, 1987; Mikulincer, 2004). Attachment styles shape one's perception of support, protection, and others' responses (Bowlby, 1973) through self‐beliefs and beliefs about others and impact on regulation strategies used to manage interpersonal closeness (Sheinbaum et al., 2015). Adult attachment research highlights anxious and avoidant attachment as two related non‐secure dimensions (Bartholomew & Horowitz, 1991). Anxious attachment bonds are thought to lead to a strong sense of negativity towards the self and search for security outside the self, intense fear of abandonment/rejection, distress, and behaviours to keep the bond and security with the attached figure (Mikulincer et al., 2003; Shaver & Mikulincer, 2002). Thus, individuals with high anxious attachment tend to describe themselves using more negative and less positive aspects (Mikulincer, 1995; Sheinbaum et al., 2015). They regulate distress through self‐devaluation and by perceiving others as similar. Avoidant attachment bonds in turn are thought to lead to a strong sense of negativity towards others, excessive self‐reliance (Gumley et al., 2014; Mikulincer, 2004) and detachment behaviours to avoid interpersonal closeness, describing the self positively (e.g., coping strategy for insecurity (Mikulincer, 2004; Sheinbaum et al., 2015)) and considering others as different (Mikulincer et al., 2003; Shaver & Mikulincer, 2002). Both anxious and avoidant attachment have been found to be elevated in individuals with psychosis (Carr et al., 2018; Korver‐Nieberg et al., 2014) and high‐risk samples (Berry et al., 2007, 2008; Russo et al., 2018). They have also been related to worse interpersonal functioning (Berry et al., 2007; de With et al., 2023; Palmier‐Claus et al., 2020; Pearse et al., 2020) and symptoms in clinical and non‐clinical populations (Berry et al., 2008; Carr et al., 2018; Gumley et al., 2014; Korver‐Nieberg et al., 2014; Strand et al., 2015; van Bussel et al., 2021). A specific role of insecure attachment in the formation of paranoia has been suggested (Carr et al., 2018; Gumley et al., 2014; Lavin et al., 2020; Murphy et al., 2020). In support of this, secure priming in experimental settings has been related to a decrease in paranoia (Newman‐Taylor et al., 2021; Sood et al., 2021). Experience sampling method (ESM) studies (Delespaul, 1995; Hektner et al., 2007) found that increased paranoia followed increased attachment insecurity (Dančík et al., 2023), in support of a causal relationship. Some studies suggest that anxious attachment has the strongest associations with paranoia (Fett et al., 2016; Korver‐Nieberg et al., 2015; Strand et al., 2015; Wickham et al., 2015); however, others suggest the strongest associations with avoidant attachment (Dančík et al., 2023; Lawrence et al., 2022).

Cognitive models of psychosis stress the implication of cognitive content and attachment‐related experiences in symptom formation (Berry et al., 2007; Freeman et al., 2002). Self‐beliefs and beliefs about others have been proposed to function as mediators of the relationship between insecure attachment and paranoia in non‐clinical (Martinez et al., 2021; Pickering et al., 2008; Sood et al., 2021) and clinical groups (Sood et al., 2022; Wickham et al., 2015), and to influence social functioning (Lavin et al., 2020; Murphy et al., 2020; Partridge et al., 2022; Sood et al., 2022). Some experimental evidence suggests that negative beliefs about others mediate the relationship between insecure attachment and paranoia (Martinez et al., 2021; Pickering et al., 2008). However, overall, evidence is still scarce and evidence on such mechanisms in daily life is lacking (Partridge et al., 2022). We therefore aimed to examine the association between self‐beliefs, beliefs about others, attachment, paranoia, and social functioning (measured as time spent alone over the course of a week). We investigated whether these associations are disorder‐specific or present across the psychosis continuum of familial risk for psychosis (Binbay et al., 2012; van Os et al., 2009), that is, in people with psychotic disorders, non‐affected first‐degree relatives, and control subjects without a family history of psychotic disorder. Studying these mechanisms over a continuum can provide valuable information regarding the shared characteristics and mechanisms that define clinical and subclinical presentations.

**FIGURE 1:**
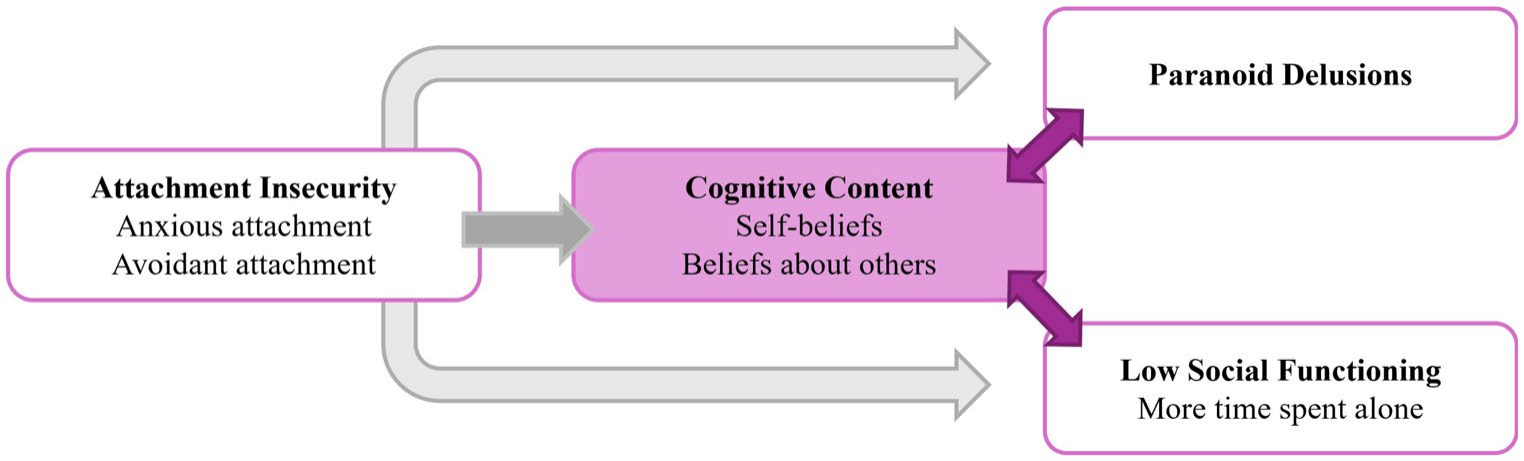
Summary of the hypothesised relationships between variables.

Since beliefs, paranoia, and social contacts are dynamic (Dančík et al., 2023; Fett et al., 2022; Lamster et al., 2017; Sheinbaum et al., 2015; Sitko et al., 2016; Thewissen et al., 2008, 2011), it is important to investigate their temporal associations. ESM allows for this in daily life (Myin‐Germeys et al., 2009), offering an ecological perspective that provides insights into temporal relationships (Palmier‐Claus et al., 2011). We used ESM to examine how self‐beliefs, beliefs about others, paranoia, and time spent alone are related in daily life and examined the relationships with attachment. A summary of the hypothesised relationships is shown in Figure 1.

We tested three primary sets of hypotheses (see Open Science Framework: https://osf.io/7g2z9). They are presented in Table 1.

**TABLE 1:** Hypotheses.

Type of comparison	Hypothesis
Group differences	Hypothesis 1: Patients show the highest attachment insecurity, paranoia, time spent alone and least positive self‐beliefs and beliefs about others; relatives occupy an intermediate position and controls show least attachment insecurity, paranoia and time spent alone and most positive self‐beliefs.
Across groups	Hypothesis 2: **(H2.1)** Higher attachment insecurity is associated with less positive self‐beliefs and beliefs about others. **(H2.2)** Higher attachment insecurity is associated with higher levels of paranoia. **(H2.3)** Less positive self‐beliefs and beliefs about others are associated with higher levels of paranoia cross‐sectionally and over time. **(H2.4)** Mediation: For anxious attachment, less positive self‐beliefs mediate the association with paranoia. For avoidant attachment, less positive beliefs about others mediate the association.
Across groups	Hypothesis 3: **(H3.1)** Higher attachment insecurity is associated with more time spent alone. **(H3.2)** Less positive self‐beliefs and beliefs about others are related to more time spent alone cross‐sectionally and over time. **(H3.3)** Mediation: For anxious attachment, less positive self‐beliefs mediate the association with time spent alone. For avoidant attachment, less positive beliefs about others mediate the association.

Abbreviation: H, hypothesis.

## MATERIALS AND METHODS

### Sample

The sample consisted of 29 patients with psychotic disorders, 17 first‐degree relatives of patients with psychotic disorders (not related to the patient participants) and 26 control subjects. The sample was recruited within the DECOP project (Decision‐making and social Context Processing in Non‐affective Psychosis). Recruitment details can be found in previous studies (Fett et al., 2022; Hanssen et al., 2020, 2022).

Inclusion criteria were age between 18 and 65, good English comprehension, >70 estimated Intelligence Quotient, and signed written consent. Exclusion criteria were history of neurological conditions and substance dependence in the six previous months of the study screening. Specific inclusion criteria for patients were having a diagnosis of a non‐affective psychotic disorder according to ICD‐10 (World Health Organization, 1992) and stable pharmacological treatment for at least 6 weeks. Specific inclusion criteria for controls were not having a psychiatric diagnosis or family history of psychosis. Ethical approval for the study was obtained from the London‐Harrow Research Ethics Committee [14/LO/0710].

### Materials

#### Sociodemographic information

Sociodemographic information (age, sex, ethnicity, living status, education, and occupation) was obtained through questionnaires.

#### Attachment insecurity

The Psychosis Attachment Measure (PAM; Berry et al., 2006) is a standardised self‐report instrument which assesses thoughts, feelings, and behaviours in close interpersonal relationships. Its 16 items are rated on a four‐point Likert scale, with anxious and avoidant attachment subscales. It shows good psychometric properties: Cronbach's alpha .82 for the anxiety dimension and .75 for the avoidance dimension in non‐clinical samples (Berry et al., 2006); .82 and .76 in clinical samples (Berry et al., 2008); and .86 and .71 in the current study, respectively.

#### Psychotic symptomatology

The Positive and Negative Syndrome Scale (PANSS; Kay et al., 1987), is a standardised instrument based on a semi‐structured interview that evaluates positive and negative symptomatology and general psychopathology through 30 items. Items are scored on a seven‐point Likert scale. The PANSS shows good psychometric properties: Cronbach's alpha .73 for the positive, .83 for the negative, and .79 for the general psychopathology scale (Kay et al., 1987); .65, .77 and .68, respectively, in the present study.

#### Experience sampling method variables

ESM was used to measure *self‐beliefs*, *beliefs about others, paranoia,* and *time spent alone*, using questions from ‘The Experience Sampling Method Item Repository’ (Kirtley et al., 2018). ESM was completed on an iPhone or iPod app over seven consecutive days, 10 times (beeps) per day at pseudo‐random times between 8:00 and 22:30, with at least 15 min and at most 1.5 h between assessments. The ESM questionnaire comprised questions regarding activities, feelings, and beliefs measured on a seven‐point Likert scale from ‘not at all’ to ‘very’ with 30 or 34 items, depending on the answer to the item ‘I am on my own’.

*Self‐beliefs* were measured with the item ‘I like myself’ (close to the concept of self‐esteem (Mruk, 2006)), as in previous studies (Sitko et al., 2016; Thewissen et al., 2008, 2011).

*Beliefs about others* were measured through a beep‐level average of the questions ‘Others trust me’, ‘In this company, I feel accepted’, ‘I like this person/these people’, ‘I feel close to this person/these people’, ‘This person/these people are dependable’, and ‘I trust this person/these people’ when people were in company of others. This structure was obtained through factor analysis, yielding a Cronbach's alpha of .95.

*Paranoia* was measured by the means of a beep‐level average of the questions ‘I feel that others dislike me’, ‘I feel suspicious’, and ‘I feel that others intend to harm me’, similar to previous ESM studies (Collip et al., 2011; Fett et al., 2022; Thewissen et al., 2008) and supported by our factor analysis, obtaining a Cronbach's alpha of .86.

*Time spent alone* was based on the question ‘I am on my own’ (yes/no). We calculated the percentage over all completed ESM questionnaires to provide an indicator of overall social functioning. To study the lagged relationship with self‐beliefs (Hypothesis 3.2), we used the variable ‘Being alone’ (0/1), based on the question ‘I am on my own’ (yes/no). See Table 2.

**TABLE 2:** Hypotheses, data level, and data analysis.

Key hypotheses	Data level for each variable	Data analysis
**(H1)** Attachment (avoidance/anxiety), time spent alone (%) over the ESM week, and paranoia are highest in patients, relatives show intermediate, and controls show the lowest scores. Patients show less positive self and other regarding beliefs than controls and relatives occupy and intermediate position.	Attachment anxiety/avoidance: participant level. Self‐beliefs and beliefs about others: observational level (ESM). Paranoia: observational level (ESM). Time spent alone (%): participant level.	General linear model Linear mixed effects model Group, age, and sex as predictors.
**(H2)** Associations between attachment, self‐beliefs and beliefs about others, and paranoia that are a prerequisite for mediation and the mediation model.		
**(H2.1)** Higher attachment insecurity is associated with less positive self‐beliefs and beliefs about others.	Attachment anxiety/avoidance: participant level. Self‐beliefs and beliefs about others: observational level (ESM).	Linear mixed effects model Self‐beliefs and beliefs about others as dependent variables and both attachment dimensions (anxious and avoidant), group and their interactions, age, and sex as predictors.
**(H2.2)** Higher attachment insecurity is related to higher levels of paranoia.	Attachment anxiety/avoidance: participant level. Paranoia: observational level (ESM).	Linear mixed effects model Paranoia as dependent variable and both attachment dimensions (anxious and avoidant), group and their interactions, age, and sex as predictors.
**(H2.3)** Less positive self‐beliefs and beliefs about others are associated with higher levels of paranoia cross‐sectionally and over time. Lagged analyses: Less positive self‐beliefs and beliefs about others at t_0_ predict higher levels of paranoia at t_1_. Vice versa, paranoia at t_0_ predicts less positive self‐beliefs and beliefs about others at t_1_.	Paranoia: observational level (ESM). Self‐beliefs and beliefs about others: observational level (ESM).	Linear mixed effects model Paranoia as dependent variable, self‐beliefs and beliefs about others, group, their interaction, age, and sex as predictors. For lagged analyses on paranoia at t_1_, self‐beliefs at t_0_ and t_1_, and paranoia at t_0_ are additionally included in the model.
**(H2.4)** Mediation models for attachment and paranoia: For anxious attachment, self‐beliefs mediate this association. For avoidant attachment, beliefs about others mediate this association.	Attachment anxiety/avoidance: participant level. Paranoia: observational level (ESM). Self‐beliefs and beliefs about others: observational level (ESM).	Mediation (Linear mixed effects model, comparison of regression coefficients and likelihood‐ratio tests) Paranoia as dependent variable, self‐beliefs and beliefs about others, attachment dimensions (anxious and avoidant), their interactions, age, and sex as predictors.
**(H3)** Associations between attachment, self‐beliefs and beliefs about others, and time spent alone that are a prerequisite for mediation and mediation model:		
**(H3.1)** Higher attachment insecurity is associated with more time spent alone (%) over the ESM week.	Attachment anxiety/avoidance: participant level. Time spent alone (%): participant level.	General linear model Time spent alone (%) as dependent variable, group, attachment dimensions (anxious and avoidant) and their interactions, age, and sex as predictors.
**(H3.2)** Less positive self‐beliefs are associated with more time spent alone over the ESM week cross‐sectionally and over time. Less positive beliefs about others are associated with more time spent alone (%) over the ESM week cross‐sectionally and over time. Lagged analyses: Being alone at t_0_ will predict less positive self‐beliefs at t_1_. Vice versa, less positive self‐beliefs at t_0_ will increase the likelihood of being alone at t_1_. Lagged analyses could not be conducted for beliefs about others which were only assessed when participants were in company of others.	Self‐beliefs and beliefs about others: observational level (ESM), relevant variables are included at both t_0_ and t_1_. For self‐beliefs and related lagged analysis: being alone (0/1) at observation level. For others‐beliefs (only assessed when in company of others): time spent alone (%) at participant level.	Linear mixed effects model (logit) Being alone (0/1) as dependent variable, self‐beliefs, group, their interaction, age, and sex as predictors. For lagged analyses on being alone at t_1_, self‐beliefs at t_0_ and t_1_, and being alone at t_0_ are additionally included in the model. General linear model Time spent alone (%) as dependent variable, beliefs about others, group, their interaction, age, and sex as predictors.
**(H3.3)** Mediation models for attachment and time spent alone: For anxious attachment, self‐beliefs will mediate this association. For avoidant attachment, beliefs about others will mediate this association.	Attachment anxiety/avoidance: participant level. Time spent alone (%): participant level. Self‐beliefs and beliefs about others: observational level (ESM).	Mediation (Linear mixed effects, comparison of regression coefficients and likelihood‐ratio tests) Time spent alone (%) as dependent variable, self‐beliefs and beliefs about others, group, attachment dimensions (anxious and avoidant), their interactions, age, and sex as predictors.

Abbreviations: %, percentage; ESM, variable measured by experience sample method; H, hypothesis.

We created lagged variables (value of the ESM variable at beep at t_0_‐t_1_) to investigate temporal associations. Lagged variables were calculated if the previous beep occurred within 180 min, as in previous studies (Bell et al., 2023).

### Procedure

All participants gave written informed consent before the beginning of the study. The study comprised two assessment sessions separated by one week. The sociodemographic questionnaire and PAM were completed during the first session. Subsequently, participants received ESM instructions (detailed explanation and demonstration). The following morning after the first session, the week of ESM data collection started. The procedure is detailed in previous studies (Fett et al., 2022; Hanssen et al., 2020, 2022).

### Data analysis

Participant's data were included in this analysis if they answered at least one‐third of the 70 possible beeps (Eisele et al., 2022; Palmier‐Claus et al., 2011; Viechtbauer, 2022). The mean number of completed ESM observations (beeps) per participant was 47.65 (*SD* = 12.73) and did not differ between groups.

All analyses were performed in R (version 4.4; R Core Team, 2024) using the R packages *esmpack* (Viechtbauer & Constantin, 2023), *lme4* (Bates et al., 2015), *lmerTest* (Kuznetsova et al., 2017), and *emmeans* (Lenth, 2024). Sample characteristics were described by means and standard deviations for quantitative variables and proportions of cases for categorical variables. Fisher's exact test was used for comparing differences in categorical variables and ANOVA to compare age differences. The specific data analysis by hypothesis is shown in Table 2.

For ESM variables, mixed multilevel regression analyses were conducted to account for the hierarchical structure of the data (multiple assessments within participants), using Kenward–Roger degrees of freedom (Kenward & Roger, 1997) for inference. In all models, we first included interactions between group (patient, relative, control) and our respective predictors of interest. Non‐significant interactions were removed from the statistical models before main effects were interpreted: non‐significant interactions suggest groups do not differ with respect to the relationships examined. Before mediation analysis, conditions for mediation were considered (Baron & Kenny, 1986). Sex and age were included in all statistical models that looked at group differences as a priori confounders. The data analysis code is available on the Open Science Framework.

## RESULTS

### Group differences (H1)

Sociodemographic data are shown in Table 3. Significant differences between groups were found for all sociodemographic variables, except for age (*F*(2, 69) = 0.549, *p* = .58). Descriptive statistics of the PAM, PANSS, and ESM variables and group comparison statistics are included in Table 4.

**TABLE 3:** Sociodemographic data.

	Controls	Relatives	Patients	*p*
*N*	26 (36.11%)	17 (23.61%)	29 (40.28%)	
Age				
*M* (*SD*)	36.15 (8.1)	37.36 (13.86)	39.07 (9.91)	.580
Sex				
Female	9 (34.6%)	11 (64.71%)	6 (20.68%)	.012
Male	17 (65.38%)	6 (35.29%)	23 (79.31%)	
Ethnicity				
Arabic	0 (0%)	1 (5.88%)	0 (0%)	< .001
Asian	7 (26.92%)	1 (5.88%)	1 (3.44%)	
Black	2 (7.69%)	5 (29.41%)	17 (58.62%)	
Indian	0 (0%)	1 (5.88%)	1 (3.44%)	
White	16 (61.54%)	8 (47.06%)	7 (24.13%)	
Other	1 (3.85%)	1 (5.88%)	2 (6.89%)	
Living status				
Alone	8 (30.77%)	4 (23.52%)	20 (68.96%)	.002
Family/partner	12 (46.15%)	10 (58.82%)	9 (31.03%)	
Other	6 (23.08%)	3 (17.64%)	0 (0%)	
Education				
None	0 (0%)	1 (5.88%)	3 (10.34%)	.038
Primary	0 (0%)	0 (0%)	2 (6.90%)	
Secondary	7 (26.92%)	0 (0%)	9 (31.03%)	
College	6 (23.07%)	5 (29.41%)	10 (34.48%)	
Other	0 (0%)	2 (11.76%)	0 (0%)	
Occupation				
Unemployed	4 (15.38%)	4 (23.53%)	22 (75.86%)	.001
Education	4 (15.38%)	3 (17.65%)	2 (6.90%)	
Employed	18 (69.23%)	10 (58.82%)	5 (17.24%)	

*Note*: *p*‐value for Age corresponds to ANOVA test, *p*‐values for other variables correspond to Fisher's exact test.

Abbreviations: *M*, mean; *N*, sample size of each group; *SD*, standard deviation.

**TABLE 4:** Means and group differences for PAM, PANSS, and ESM variables.

	Controls	Relatives	Patients	Main effect interaction	Group comparisons
*n*	26	17	29		
Attachment anxiety	1.96 (.62)	2.04 (.80)	2.05 (.78)	*F*(2, 67) = 0.11; *p* = .895	
Attachment avoidance	1.48 (.47)	1.58 (.64)	1.57 (.54)	*F*(2, 67) = 0.29; *p* = .752	
Positive symptoms			13.00 (4.21)		
PANSS P6 (suspiciousness)			2.68 (1.22)		
Negative symptoms			15.21 (5.82)		
General psychopathology			27.33 (5.66)		
Paranoia	1.53 (.91)	1.71 (.75)	2.24 (1.25)	*F*(2, 67) = 3.03; *p* = .055	Con‐Rel: *b* = −0.23, *SEM* = 0.34, *t*(67.1) = −0.69, *p* = .771 Con‐Pat: *b* = −0.70, *SEM* = 0.29, *t*(66.9) = −2.44, *p* = .045 Rel‐Pat: *b* = −0.47, *SEM* = 0.34, *t*(67.1) = −1.37, *p* = .363
Self‐beliefs	5.05 (.96)	5.13 (1.47)	5.21 (1.32)	*F*(2, 67) = 0.05; *p* = .953	
Beliefs about others	5.26 (1.17)	5.23 (1.19)	5.38 (.88)	*F*(2, 67) = 0.31; *p* = .737	
Percentage time alone	57.21 (24.34)	44.66 (21.91)	69.53 (30.34)	*F*(2, 67) = 5.21; *p* = .008	Con‐Rel: *b* = 7.88, *SEM* = 8.22, *t*(67) = 0.96, *p* = .605 Con‐Pat: *b* = −9.14, *SEM* = 7.01, *t*(67) = −1.30, *p* = .398 Rel‐Pat: *b* = −17.02, *SEM* = 8.36, *t*(67) = −2.04, *p* = .111
Completed ESM beeps	51.15 (12.54)	42.24 (11.67)	47.69 (12.8)	*F*(2,67) = 2.85; *p* = .065	

*Note*: For each variable from each study group, means are presented with standard deviations in parentheses. *n* = sample size of each group; attachment anxiety and avoidance scores come from the Psychosis Attachment Measure (Berry et al., 2006); *F* = *F* statistic for group effect with degrees of freedom in parentheses; *p* = *p* value; positive and negative symptoms and general psychopathology correspond to the subscales of the Positive and Negative Syndrome Scale (PANSS; Kay et al., 1987); PANSS P6 corresponds to item number 6 of the PANSS (Kay et al., 1987); no scores are shown for controls and relatives for the PANSS, because the PANSS was only administered to patients; beliefs about the self, beliefs about others, paranoia, and percentage of time alone correspond to ESM variables; Con‐Rel = post hoc comparison between control and relative groups; Con‐Pat = post hoc comparison between control and patient groups; Rel‐Pat = post hoc comparison between relative and patient groups; *b* = estimate of the linear model; *SEM* = standard error of the mean; *t = t* statistic for pairwise comparisons with degrees of freedom in parentheses.

The groups did not differ significantly in anxious or avoidant attachment, self‐beliefs and beliefs about others. There was a marginally significant group difference in paranoia, where patients showed higher levels than controls. Also, there was a significant group difference in terms of percentage of time spent alone, but differences between individual groups remained non‐significant (Table 4).

### Attachment, self‐beliefs, beliefs about others, and paranoia (H2)

#### Attachment, self‐beliefs, and beliefs about others (H2.1)

Interactions between insecure attachment and group on self‐beliefs and beliefs about others were non‐significant and consequently removed from the models. In the models without interaction, anxious attachment was negatively and significantly related to self‐beliefs (*b* = −0.48, *SEM* = 0.20, *t*(66.84) = −2.45, *p* = .017): beliefs were less positive with more anxious attachment. The association between avoidant attachment and self‐beliefs was also negative and close to statistical significance (*b* = −0.52, *SEM* = 0.26, *t*(66.97) = −1.98, *p* = .052). There were no significant associations between either insecure attachment dimension and beliefs about others (anxious: *b* = −0.06, *SEM* = 0.18, *t*(64.30) = −0.35, *p* = .730; avoidant: *b* = −0.34, *SEM* = 0.24, *t*(64.86) = −1.42, *p* = .161).

#### Attachment and paranoia (H2.2)

Interactions between insecure attachment dimensions and group were non‐significant and consequently removed from the models. In the models without interactions, significant positive associations were present between anxious (*b* = 0.78, *SEM* = 0.15, *t*(66.86) = 5.08, *p* < .001) and avoidant attachment (*b* = 0.41, *SEM* = 0.20, *t*(66.97) = 2.01, *p* = .048) and paranoia.

#### Self‐beliefs, beliefs about others, and paranoia—cross‐sectional and lagged associations (H2.3)

*Self‐beliefs* were negatively and significantly associated with paranoia cross‐sectionally (*b* = ‐0.13, *SEM* = 0.01, *t*(3.42) = −10.24, *p* = .001). However, there were no significant temporal associations: self‐beliefs at t_0_ did not predict paranoia over time (i.e., at t_1_, *b* = −0.0020, *SEM* = 0.0014, *t*(2590) = −1.35, *p* = .176) and, reversely, paranoia at t_0_ did not predict self‐beliefs over time (i.e., at t_1_ *, b* = −0.033, *SEM* = 0.03, *t*(2591.99) = −1.29, *p* = .199).

*Beliefs about others* were significantly related with paranoia cross‐sectionally (*b* = −0.10, *SEM* = 0.01, *t*(1367) = −7.639, *p* < .001). Beliefs about others at t_0_ did not predict paranoia over time (i.e., at t_1_, *b* = −0.01, *SEM* = 0.02, *t*(731.15) = −0.64, *p* = .522) and, reversely, paranoia at t_0_ did not predict beliefs about others over time (i.e., at t_1_, *b* = 0.11, *SEM* = 0.07, *t*(720.11) = 1.52, *p* = .128).

#### Mediation models—Attachment, paranoia, and self‐beliefs and beliefs about others as mediators (H2.4)

##### Self‐beliefs as mediator

Associations between self‐beliefs, paranoia, and insecure attachment dimensions were significant and a mediation model was run. Including self‐beliefs as a mediator reduced the effect size of the associations between anxious and avoidant attachment and paranoia slightly, and the association with avoidant attachment was no longer significant (anxious: *b* = 0.68, *SEM* = 0.15, *t*(65.15) = 4.63, *p* < .001; avoidant: *b* = 0.30, *SEM* = 0.19, *t*(65.21) = 1.56, *p* = .124). The comparison of the simple and mediation model for anxious attachment through a likelihood‐ratio test showed a better model fit of the mediation model (AIC_nomediation_ = 6586.4; AIC_mediation_ = 6476.6; *χ* ^*2*^(5) = 119.88, *p* < .001).

##### Beliefs about others as mediator

In the absence of significant associations between insecure attachment dimensions and beliefs about others, a mediation analysis was not conducted.

### Attachment, self‐beliefs, beliefs about others, and social functioning (H3)

#### Attachment and time spent alone (H3.1)

Interactions between group and insecure attachment dimensions on percentage of time spent alone were not significant and were consequently removed from the model. In models without the interaction terms, associations between insecure attachment and percentage of time spent alone were non‐significant (anxious: *b* = 1.85, *SEM* = 4.39, *t*(65) = 0.42, *p* = .676; avoidant: *b* = 7.83, *SEM* = 5.79, *t*(65) = 1.35, *p* = .181).

#### Self‐beliefs, beliefs about others, and time alone/being alone—cross‐sectional and lagged associations (H3.2)

*Self‐beliefs* were negatively and significantly associated with being alone cross‐sectionally (*b* = −0.14, *SE* = 0.05, *z* = −2.91, *p* = .004). There was no significant temporal association between self‐beliefs at t_0_ and the likelihood of being alone at the later time point t_1_ (*b* = 0.003, *SE* = 0.06, *z* = 0.046, *p* = .96). However, being alone at t_0_ predicted self‐beliefs at t_1_ (*b* = −0.075, *SEM* = 0.04, *t*(2538.42) = 1.989, *p* = .047).

*Beliefs about others* negatively and significantly predicted the overall percentage of time spent alone (*b* = −7.36, *SEM* = 1.29, *t*(2590) = −5.70, *p* < .001). We could not test a temporal relationship, because participants were asked about beliefs about others only when they were not alone, i.e. only the percentage of time spent alone over the ESM week was available for analysis.

#### Mediation models—Attachment dimension, time spent alone, and self‐beliefs and beliefs about others as mediators (H3.3)

Given the non‐significant findings regarding attachment insecurity and time alone (3.3.1), no mediation models were run.

## DISCUSSION

This ESM study examined the role of self‐beliefs and beliefs about others in the relationship between insecure attachment, paranoia, and social functioning across a continuum of psychosis risk. Our most important findings supports the relationships between attachment insecurity, less positive self‐ and other‐regarding beliefs, and paranoia that exist across the psychosis continuum. However, only cognitions about the self, but not others, mediated the association between attachment insecurity and paranoid beliefs. Unexpectedly, attachment insecurity was unrelated to the amount of social contact our participants had in daily life. However, people who were more often alone held less positive beliefs about themselves and others, and being alone at a specific time was related to less positive self‐beliefs. Our findings support an attachment‐based treatment approach for paranoia that focuses on self‐ and other‐regarding beliefs and their relationship with social interaction.

As expected, we found that patients had higher levels of paranoia than controls (Pinkham et al., 2012), with relatives occupying an intermediate position, congruently with the hypothesised continuum (Freeman et al., 2005, 2010; Hajdúk et al., 2019; Johns & van Os, 2001; van Os et al., 2009). Patients were alone most often, although group differences did not reach significance (Fett et al., 2022; Pinkham et al., 2012; Velthorst et al., 2017). Results contradicted our hypothesised group differences for attachment and self‐ and other‐regarding beliefs. The absent group differences in attachment insecurity are in line with previous findings (Bakermans‐Kranenburg & van Ijzendoorn, 2009; Berry et al., 2007, 2008; Carr et al., 2018; Korver‐Nieberg et al., 2014, 2015; Russo et al., 2018; Varela et al., 2021). However, they are at odds with many studies that report such differences (Carr et al., 2018; de With et al., 2023). Discrepancies could be related to the nature of the samples or the used measures. The PAM (Berry et al., 2006) is widely used and has good psychometric properties, yet it only assesses insecure attachment dimensions. Significant differences may appear when evaluating attachment security.

Regarding self‐beliefs, many studies reported worse self‐evaluations in patients with psychotic disorders than in controls (Dickson et al., 2016; Frank & Davidson, 2012; Kesting et al., 2011; Penn et al., 2000; Yanos et al., 2008). In our study, in contrast, participants in all three groups evaluated themselves on average equally positively in response to the statement ‘I like myself’. Similar, positive ratings have been reported using the questions ‘I like myself’ and ‘I doubt myself’, although group differences were significant in the much larger sample (Daemen et al., 2022). Possible explanations for the divergent findings could be differences in measures employed: standardised instruments, for example, the Rosenberg Self‐Esteem Scale (Rosenberg, 1979), provide more comprehensive assessments of self‐esteem through questions that look at positive and negative self‐beliefs (e.g., ‘At times I think I am no good at all’, ‘I certainly feel useless at times’ (Combs & Penn, 2004; Kesting et al., 2011; Yanos et al., 2008)). Other ESM studies used slightly more complex measures, including additional statements as ‘I am a good person’, ‘I am ashamed of myself’, and ‘I am a failure’ (Thewissen et al., 2008, 2011). Thus, our question ‘I like myself’ could have differentiated groups less than more complex and/or explicit probes of self‐beliefs (Fowler et al., 2006; Mruk, 2006). In addition, it has been proposed that individuals with psychosis, and paranoid delusions in particular, may have equivalent levels of explicit self‐esteem compared to healthy controls, but that they have lower implicit self‐esteem (Bentall et al., 2001; Mackinnon et al., 2011; Valiente et al., 2011). Thus, positive explicit self‐beliefs as measured here may reflect defensive patterns (Greenwald & Banaji, 1995) and different results could emerge for implicit self‐esteem (Greenwald & Farnham, 2000).

Finally, while patients in our sample had fewer social interactions, their beliefs about others suggested that they still viewed their more limited social contacts positively. Contrary to our hypotheses and previous research, beliefs about others did not differ between groups (Fowler et al., 2006; Shen et al., 2021). Importantly, although our composite measure achieved high internal consistency, all our questions regarding beliefs about others were phrased positively, and it is possible that different findings emerge for explicit negative beliefs about others (e.g., ‘Others cannot be trusted, are nasty or bad’ (Fowler et al., 2006; Levenson, 1974)). Furthermore, it is important to note that the current sample was clinically relatively well, which might account for some differences in findings (Fowler et al., 2006).

Our first key research question concerned the relationship between attachment insecurity, beliefs, and paranoia. We found that, regardless of group, more anxiously attached individuals expressed they liked themselves less. This finding is consistent with regulatory strategies anxiously attached individuals use and the type of cognitive content characterised by a strong sense of negativity and negative self‐views (Mikulincer, 2004; Shaver & Mikulincer, 2002), in line with previous research (Berry et al., 2006; Martinez et al., 2021; Pickering et al., 2008; Sheinbaum et al., 2015; Sood et al., 2022; Wickham et al., 2015). In contrast to our hypotheses, higher levels of avoidant attachment were also related to more negative self‐beliefs rather than more positive beliefs. This also has been reported by others (Berry et al., 2006; Sheinbaum et al., 2015). It contradicts the idea that avoidantly attached people use positive self‐descriptions (Martinez et al., 2021) as a strategy to cope with their real insecurity and negative self‐appraisal (Mikulincer, 1995; Ponizovsky et al., 2013; Sheinbaum et al., 2015). However, in our study, participants did not answer the questions in negative social situations. Thus, it is possible that detachment behaviours, defensive responses, and subsequent self‐enhancement strategies remained inactivated.

In addition, contrary to our expectations, individuals’ beliefs about others were not significantly related to attachment insecurity, which contrasts with theory and previous research findings (Mikulincer, 2004; Shaver & Mikulincer, 2002). Others (Wickham et al., 2015) also failed to find significant associations between insecure attachment dimensions and beliefs in powerful others (i.e., external locus of control (Levenson, 1974)). A tend‐level increase in negative beliefs about others in response to primed anxious and avoidant attachment has been found (Sood et al., 2021). However, the study assessed explicitly negative beliefs. Other ESM research (Sheinbaum et al., 2015) showed that individuals with insecure attachment styles felt less cared for and less close to others. Discrepancies with these findings might be explained by different operationalisations of beliefs about others. Further, it is possible that beliefs about others may have to be activated through a situation. We did not distinguish who participants were with, and the expression of attachment styles in social situations may depend on the level of closeness (Sheinbaum et al., 2015). Further investigations should test the importance of different types of beliefs (e.g., lower positive vs. higher negative) and whether associations exist for specific persons and/or relationships.

Other important findings are the significant positive associations between the insecure attachment dimensions and paranoia, consistent with previous literature in clinical and non‐clinical samples (Fett et al., 2016; Murphy et al., 2020; Pickering et al., 2008; Ponizovsky et al., 2013; Wickham et al., 2015). In line with others, we found a much stronger association between anxious attachment and paranoia than between avoidant attachment and paranoia (Fett et al., 2016; Korver‐Nieberg et al., 2015; Lavin et al., 2020; Strand et al., 2015; Wickham et al., 2015). Stronger associations between anxious attachment and paranoia may be explained by increased worry, negative affect, and social threat perception (Freeman et al., 2002, 2014; Mikulincer & Shaver, 2012): anxiously attached people may increase hypervigilance and control as strategies to deal with potential threats in relationships (Mikulincer et al., 2003; Shaver & Mikulincer, 2002), which may trigger paranoid thoughts about others. According to the threat anticipation cognitive model (Freeman et al., 2002), sustained worry, negative affect towards relationships, high interpersonal sensitivity, and negative self‐beliefs may lead individuals to interpret behaviours in relationships as threatening, thereby developing paranoid explanations of others’ actions (Freeman et al., 2014; Orth et al., 2022).

Furthermore, as expected (Freeman et al., 2002; Garety et al., 2001, 2007) and consistently reported (Daemen et al., 2022; Jones et al., 2010; Kesting et al., 2013; Lamster et al., 2017; Sood et al., 2021; Thewissen et al., 2008, 2011), higher levels of paranoia were significantly related to more negative self‐beliefs and beliefs about others, with similar effect sizes across groups (Kesting & Lincoln, 2013; Lüdtke et al., 2023; Tiernan et al., 2014). These relationships were only found for concurrent associations, but not over time. This result adds to a body of contradicting findings regarding temporal associations between beliefs and paranoia in daily life (Lüdtke et al., 2023). It suggests that the interdependence between self‐ and other‐related beliefs and paranoia happens concurrently in the moment, where they feedback on each other in a specific situation/context. It is also possible that this association is explained by other factors, such as negative mood or distress (Lüdtke et al., 2023). Importantly, other ESM research which used a slightly different measure of self‐esteem in a larger sample did report significant bi‐directional temporal associations between self‐beliefs and paranoia (Daemen et al., 2022). Yet, it is unclear whether their lagged analyses controlled for the respective variables at the same point (reducing lagged effects), highlighting the need for further carefully controlled investigations that can show under which circumstances and in which individuals these associations emerge.

Consistent with expectations, less positive self‐beliefs mediated the association between both dimensions of attachment insecurity and paranoia; however, this effect was only partial for anxious attachment (Martinez et al., 2021; Pickering et al., 2008; Sood et al., 2021; Wickham et al., 2015). These findings held across groups, highlighting the importance of attachment and self‐related cognitive content for suspiciousness in daily life as a phenomenon that is not limited to clinical psychosis. ESM research further shows associations between early traumatic experiences, insecure attachment (Gabínio et al., 2018; Garety et al., 2007; Yumbul et al., 2010) and paranoia are mediated by negative self‐beliefs in patients, at risk‐groups and controls (Daemen et al., 2022). This finding may extend to other ESM results where attachment style moderates the relationship between social proximity seeking and paranoia (Lawrence et al., 2022): for individuals with high avoidant attachment, help‐seeking behaviours may activate negative self‐beliefs following the social contact—experienced as aversive—intensifying paranoid beliefs, congruent with attachment theory (Mikulincer, 2004; Mikulincer et al., 2003; Shaver & Mikulincer, 2002; Sheinbaum et al., 2015). In sum, findings suggest a tendency in insecurely attached people to perceive themselves less positively, which may fuel thoughts about being harmed or mistreated by others. Our findings support cognitive models of psychosis (Freeman et al., 2002) and suggest that considering insecure attachment and self‐beliefs offers important therapeutic opportunities to ameliorate paranoid beliefs.

Our second key research question concerned the relationship between attachment insecurity, beliefs, and social functioning, operationalised as time spent alone. We did not find any significant associations between insecure attachment and time spent alone, contrasting earlier work (de With et al., 2023). The differences in findings might be due to different conceptualisations of social functioning, where others (de With et al., 2023; Sheinbaum et al., 2015) assessed pro‐social behaviour, social withdrawal, interpersonal behaviour, or social appraisals. Time spent with others includes numerous social interactions (e.g., family, work, or care contexts), which differ in quality and aspects of social motivation (e.g., which is not needed for social contact in work or clinical contexts). The inclusion of non‐chosen social contacts, as well as non‐bonded ones, in our conceptualisation may explain why the percentage of time alone was not related to attachment insecurity.

Concerning relationships between beliefs and time spent alone, being alone was associated with more negative self‐beliefs at the same time (i.e., same ESM beep). Positive effects of social interaction on concurrent self‐beliefs may be explained by changes in affect or stress levels (Daemen et al., 2022; Thewissen et al., 2011). Being alone rather than in company at a specific moment was also related to a higher likelihood of more negative self‐beliefs later on. This could point toward the positive impact of social interaction on self‐beliefs, as suggested by others who found that pursuing social and interpersonal activities had positive effects on self‐esteem (Frank & Davidson, 2012). Contrastingly, we did not find that self‐beliefs predicted the likelihood of engaging in social interactions at a later point in time. Finally, a higher percentage of overall time spent alone over the ESM week was related to less positive beliefs about others. It is possible that those beliefs may reduce the desire for social contact, or vice versa, that lacking social contacts could drive these beliefs about others (Fett et al., 2022).

### Clinical implications

Our findings imply that how individuals view themselves can be an important therapeutic goal in approaches that aim to address suspiciousness and paranoid delusions, e.g., within recommended treatments for psychotic disorders (National Institute for Health and Care Excellence, 2014), but also in interventions for populations with different levels of suspiciousness and paranoid experiences, which may be offered in daily‐life contexts. Emerging evidence suggests that these approaches can enhance self‐esteem with positive downstream effects on general psychopathology and quality of life (Freeman et al., 2014; Reininghaus et al., 2024). Additionally, our findings indicate that self‐beliefs should be considered as a reflection of inner models about the world, related to attachment experiences (Palmier‐Claus et al., 2020). The observed associations between less positive beliefs about others, suspiciousness, and more time spent alone across groups underscore the important role of such cognitive content in reducing paranoid beliefs and promoting social engagement in different treatment contexts to achieve better recovery outcomes (van Bussel et al., 2021), highlighting their relevance for cognitive interventions (Chadwick et al., 1996; Fowler et al., 1995; National Institute for Health and Care Excellence, 2014).

Thus, self‐ and other‐regarding beliefs should be therapeutic targets. Addressing them through cognitive restructuring and the modelling of new social behaviours (Reininghaus et al., 2024) may promote positive self‐ and other‐regarding beliefs and increase positive social engagement.

Lastly, our findings support the utility of attachment‐based CBT models for psychosis (Newman‐Taylor et al., 2022). These models address how attachment relates to cognitive, behavioural, and affective processes, so that maladaptive interpersonal patterns can be recognised and reflected upon. Thus, guiding more effective staff–patient interactions to facilitate recovery in psychosis.

### Limitations and future perspectives

Our findings need to be considered in the light of several limitations. Patients were relatively clinically stable; thus, the current findings may not generalise to patients in acute states of psychosis. Besides, although this study employed ESM questions that have been extensively used (Viechtbauer, 2022), these do not constitute acomprehensive measure of the constructs under investigation. This might explain some of the contradictory findings. Future research should consider the inclusion of additional validated measures to assess beliefs and social functioning. Further, the construct beliefs about others’ was robust from a psychometric perspective, yet it did not capture explicitly negative beliefs about others. Further research should examine the relative importance of reduced positive and increased negative other‐regarding beliefs (e.g., mistrust, dependency).

## CONCLUSIONS

We found that particularly beliefs about the self were important in the relationship between insecure attachment and paranoia. Our study is one of the first to simultaneously examine the relative impact of other‐regarding beliefs in the association between attachment and paranoia. While the results did not show a mediating role, less positive beliefs about others were related to paranoid thinking and less social contact across the psychosis continuum. This suggests the clinical utility of a therapy focus on attachment, self‐beliefs, and beliefs about others in a variety of therapeutic and clinical settings to exert beneficial effects on paranoid thinking and social engagement.

## AUTHOR CONTRIBUTIONS

**Pilar de‐la‐Higuera‐Gonzalez:** Conceptualisation; methodology; data curation; formal analysis; visualisation; writing – original draft; writing – review and editing. **Carsten Allefeld:** Methodology; data curation; formal analysis; writing – review and editing. **Alejandro de la Torre‐Luque:** Writing – review and editing. **Ana Isabel Guillén:** Writing – review and editing. **Marina Díaz‐Marsá:** Writing – review and editing. **Anne Kathrin Fett:** Conceptualisation; methodology; data curation; investigation; formal analysis; funding acquisition; visualisation; writing – original draft; writing – review and editing.

## CONFLICT OF INTEREST STATEMENT

On behalf of all authors, the corresponding author states that there is no conflict of interest.

## Data Availability

Research data are not shared, due to the fact that sharing data compromises ethical standards and legal requirements. However, the scripts used to generate the analyses presented in the study, as well as the resulting outputs, are publicly archived in the OSF public repository: https://osf.io/s5b2c?view_only=eef7e5fa1f3c489784cbdbc885d7f9d3.
